# Intrinsic and Extrinsic Charge Transport in CH_3_NH_3_PbI_3_ Perovskites Predicted from First-Principles

**DOI:** 10.1038/srep19968

**Published:** 2016-01-29

**Authors:** Tianqi Zhao, Wen Shi, Jinyang Xi, Dong Wang, Zhigang Shuai

**Affiliations:** 1 MOE Key Laboratory of Organic OptoElectronics and Molecular Engineering, Department of Chemistry, Tsinghua University, Beijing 100084, P.R. China; 2 Key Laboratory of Organic Solids, Beijing National Laboratory for Molecular Science (BNLMS), Institute of Chemistry, Chinese Academy of Sciences, Beijing 100190, P.R. China; 3 Collaborative Innovation Center of Chemistry for Energy Materials, Xiamen University, 351005 Xiamen, China

## Abstract

Both intrinsic and extrinsic charge transport properties of methylammonium lead triiodide perovskites are investigated from first-principles. The weak electron-phonon couplings are revealed, with the largest deformation potential (~ 5 eV) comparable to that of single layer graphene. The intrinsic mobility limited by the acoustic phonon scattering is as high as a few thousands cm^2^ V^−1^ s^−1^ with the hole mobility larger than the electron mobility. At the impurity density of 10^18^ cm^−3^, the charged impurity scattering starts to dominate and lowers the electron mobility to 101 cm^2^ V^−1^ s^−1^ and the hole mobility to 72.2 cm^2^ V^−1^ s^−1^. The high intrinsic mobility warrants the long and balanced diffusion length of charge carriers. With the control of impurities or defects as well as charge traps in these perovskites, enhanced efficiencies of solar cells with simplified device structures are promised.

Organolead trihalide perovskites represent a novel class of materials for solar energy conversions^1^. The rapid boost in power conversion efficiencies of perovskites based solar cells^2^ has triggered enormous investigations towards understanding the fundamental properties of these materials^3^,^4^,^5^,^6^,^7^,^8^. The charge carrier diffusion length in solution-grown single crystals was shown to exceed 100 micrometers^3^. The trap density on the order of 10^10^ cm^−3^ was estimated^3^,^4^, and the trap-free mobility up to a hundred and a few tens of cm^2^ V^−1^ s^−1^ was derived for holes and electrons, respectively^3^. The studies on exciton and charge carrier dynamics by transient absorption, fluorescence lifetime, time-resolved microwave conductivity and other experimental techniques have greatly deepened our understanding of the fundamental aspects of hybrid perovskites^3^,^4^,^5^,^6^,^7^,^8^. Nonetheless, the material properties derived experimentally rely heavily on the fabrication process of materials. For instance, the charge carrier diffusion length in single crystals of perovskites is three orders of magnitude greater than that in polycrystalline films^3^,^4^,^5^,^6^. In fact, direct measurement of charge transport properties is not available until recently with the first successful fabrication of lead iodide perovskite based field-effect transistors^9^,^10^. The transistors exhibited balanced ambipolar transport with mobilities of ~10^−2^ cm^2^ V^−1^ s^−1^ at 78 K^9^ and 1 cm^2^ V^−1^ s^−1^ at room temperature^10^ respectively. Considering the inconsistency in the transport parameters reported, the first-principles modeling of charge transport properties of perovskites is urgently needed, which will help understand the superior optoelectronic properties of CH_3_NH_3_PbI_3_, and aid in the design of more efficient and environmentally-friendly photovoltaic materials. Indeed, the electronic structure and band gaps of organic-inorganic hybrid perovskites have been intensively studied by the density functional theory (DFT) methods^11^,^12^,^13^,^14^,^15^,^16^. Theoretical calculations also revealed that this unique family of material is tolerant to defects, because the intrinsic point defects do not generate gap states that act as trap centers for charge carriers^17^,^18^,^19^. As a result, Shockley-Read-Hall recombination is greatly suppressed. Nonetheless, these defects can act as scattering centers, and play a significant role in charge transport of hybrid perovskites if abundant defects or impurities exist in the fabricated materials. In this work, we aim to uncover both intrinsic and extrinsic charge transport properties of CH_3_NH_3_PbI_3_ based on first-principles calculations, by incorporating both acoustic phonon and charged impurity scattering mechanisms. We have revealed relatively weak electron-phonon couplings in perovskites, with the largest deformation potential (~ 5 eV) comparable to that of single layer graphene. The hole mobility limited by the acoustic phonon scattering is larger than the electron mobility, and both are up to a few thousands cm^2^ V^−1^ s^−1^. The charged impurity scattering starts to dominate at the impurity concentration of 10^18^ cm^−3^, and it lowers the electron mobility to 101 cm^2^ V^−1^ s^−1^ and the hole mobility to 72.2 cm^2^ V^−1^ s^−1^. The high intrinsic mobility warrants the long and balanced diffusion length of charge carriers. By defects and interface engineering, enhanced efficiencies of perovskites-based solar cells with simplified device structures are promised.

## Results

**Figure 1:**
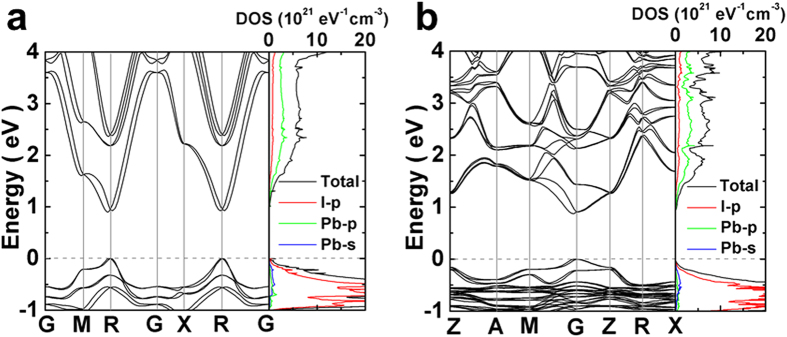
Band structure and partial density of states. (**a**) Cubic CH_3_NH_3_PbI_3_. (**b**) Tetragonal CH_3_NH_3_PbI_3_.

Both room temperature tetragonal and high temperature cubic phases of CH_3_NH_3_PbI_3_ were studied. It has been shown that the simultaneous incorporation of spin-orbit coupling (SOC) effect and many-body effect can deliver a balanced description of band gaps for Pb- and Sn-based perovskites^14^,^15^. In this work, we took into account the SOC effect in the calculation of band structures, while applied a scissor operator to the band energies during the calculation of charge transport properties to compensate the underestimated band gap by SOC-DFT. Specifically, the energies of conduction bands were shifted upwards to reproduce the experimental band gap of 1.6 eV^20^. In the cubic phase, the methylammonia cation was oriented along the [100] direction, since earlier studies suggested that such orientation led to a relatively lower energy^15^. The crystal structure of tetragonal phase was taken from Ref. 20, with the organic cation oriented in the [001] direction. The lattice parameters and atomic positions were fully optimized using the Perdew-Burke-Ernzerhof (PBE) functional^21^ within the generalized gradient approximation (GGA), as implemented in the Vienna *ab initio* simulation package (VASP)^22^,^23^. After optimization, the cubic lattice became pseudocubic, with a profound elongation along the [100] direction. Both cubic and tetragonal phases of CH_3_NH_3_PbI_3_ are direct band gap semiconductors. The band gap of the cubic phase opens at the R point, and that of the tetragonal phase opens at the Γ point (Fig. 1). The band gaps were severely underestimated by SOC-DFT, as has been demonstrated in previous studies^14^,^15^. The partial density of states analysis (Fig. 1) showed that the valence band maximum (VBM) arises predominantly from the 5p-orbital of I with a small contribution from the 6s-orbital of Pb, and the conduction band maximum (CBM) is constituted by the 6p-oribtal of Pb and 5p-orbital of I. The orbitals of methylammonium cations are localized with deep energy levels, so they do not contribute to the frontier orbitals that are responsible for charge transport.

**Figure 2:**
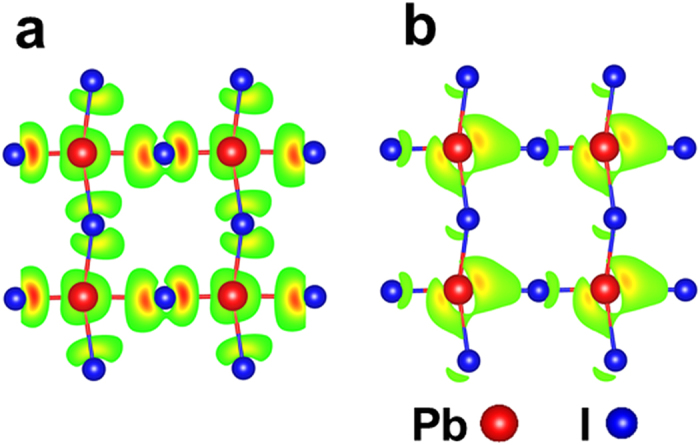
Electron density at the R point of cubic CH_3_NH_3_PbI_3_. (**a**) VBM. (**b**) CBM.

Similar to inorganic semiconductors, charge carriers in organolead perovskites are delocalized and a bandlike transport mechanism is anticipated. The electrons and holes are scattered by phonons, impurities or defects when they are accelerated by an electric field, and their mean free paths are shortened due to these scattering events. The scattering by longitudinal acoustic phonons is well modeled by the deformation potential (DP) theory, and the scattering matrix elements can be easily extracted from first-principles. We are among the first to apply the DP theory to predicting the charge carrier mobility in graphene and other carbon allotropes^24^,^25^,^26^,^27^, and the approach has now been widely recognized to model charge transport in novel low-dimensional materials. To extract the scattering matrix elements, the crystal was strained along three crystallographic axes respectively to mimic the longitudinal acoustic phonons propagating in these directions. The total energy change with respect to the strain produces the elastic constant *C*_*ii*_ via 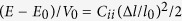, and the shift of CBM and VBM due to the dilation yields the deformation potential 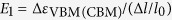 of electrons and holes. In bulk materials lacking of vacuum, it is essential to calibrate the band energies during straining by inner energy levels^28^. Here, we took the deep energy level of organic cations as the reference, and reasonably assumed that its position was not influenced by lattice deformations (Supplementary Fig. S1). The deep core level calibration method is certainly not rigorous, but it is easy to apply and does not require additional calculations. We noticed that there is a report on calculating the absolute volume deformation potential independent of the selection of the reference energy levels^29^. Actually, we have checked the deformation potentials derived from two core levels, and obtained almost the same results. The elastic constants of perovskites are anisotropic (Table 1), consistent with their crystal structures (Supplementary Table S1). For example, in the pseudocubic phase the lattice is more easily strained in the elongated [100] direction. The deformation potential of electrons is larger than that of holes (Table 1), indicating that electrons are more strongly scattered by lattice vibrations than holes. The electron density contour plotted in Fig. 2 showed that the 6s-orbital of Pb and 5p-orbital of I constituting the VBM are centered on the atomic nuclei, exhibiting an ionic bond character. The 6p-orbital of Pb and 5p-orbital of I forming the CBM exhibit an antibonding character, so its energy is more prone to change when subject to lattice deformations. Overall, the electron-phonon couplings in CH_3_NH_3_PbI_3_ are relatively weak, with the largest deformation potential of ~ 5 eV, comparable to that of single layer graphene^24^,^25^.

**Figure 3:**
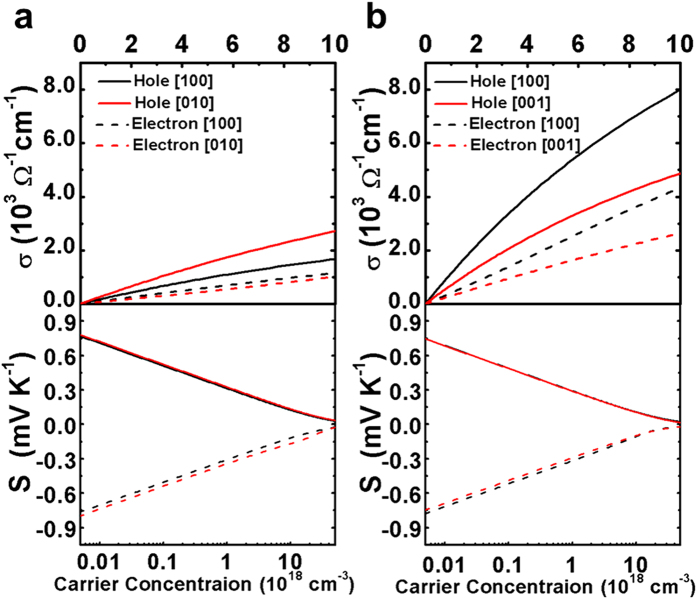
Electrical conductivity and thermopower as a function of carrier concentration at 300 K. (**a**) Cubic CH_3_NH_3_PbI_3_. (**b**) Tetragonal CH_3_NH_3_PbI_3_.

The semiclassical Boltzmann transport theory in the relaxation time approximation was subsequently applied to describe charge transport in CH_3_NH_3_PbI_3_. The band energies on a much dense **k**-mesh were calculated based on the converged charge density, and interpolated on a **k**-mesh ten times denser. The convergence of transport coefficients with respect to the **k**-point sampling has been tested. The Fermi-integral and electrical transport coefficients were calculated with the BoltzTraP package^30^, in which we have incorporated the relaxation time calculations based on the DP theory with the scattering matrix element being 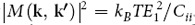. Due to the weaker charge-acoustic phonon interactions, the average relaxation time of holes is five times larger than that of electrons, both are on the order of picoseconds (Table 1). Correspondingly, the mean free path of electrons and holes falls in the range from a few tens to a few hundreds of nanometers (Table 1). The room temperature electrical conductivity and Seebeck coefficient as a function of the carrier concentration were shown in Fig. 3. At low carrier densities, the electrical conductivity increases linearly with the carrier concentration, and the slope of the curve gives the charge carrier mobility. We have found that the hole mobility is larger than the electron mobility for both cubic and tetragonal phases of CH_3_NH_3_PbI_3_, due to the weaker phonon scattering effect and the larger relaxation time of holes. This finding has some experimental supports: the 9 GHz mobility measured by the time-resolved microwave conductivity (TRMC) was separated to 17 cm^2^ V^−1^ s^−1^ for holes and 3 cm^2^ V^−1^ s^−1^ for electrons^7^, and the trap-free space charge limit current (SCLC) mobility of 164 cm^2^ V^−1^ s^−1^ and 24.8 cm^2^ V^−1^ s^−1^ was derived for holes and electrons respectively^3^. The charge carrier mobility measured by different methods on different samples is usually quite different, but the magnitude falls consistently in the range from a few tens to a few hundreds of cm^2^ V^−1^ s^−1^ 3,4,7,8,20. Our calculations reveal that the intrinsic charge carrier mobility limited by the acoustic phonon scattering can be as high as a few thousands of cm^2^ V^−1^ s^−1^. We noticed that a previous study of the cubic CH_3_NH_3_PbI_3_ based on the effective mass approximation and DP theory reported charge carrier mobilities on the same order of magnitude as our prediction, but the electron mobility was larger than the hole mobility^15^. The difference seems to originate primarily from the deformation potential, whose value may be sensitive to the calibration method. As mentioned above, for bulk materials lacking of vacuum the band energies calculated during straining have to be carefully calibrated. We took the deep energy level of organic cations as the reference, and the similar calibration method has been applied to extracting the work function of perovskites^16^. In our case, the deformation potential of holes is smaller than that of electrons so that the hole mobility is larger than the electron mobility, even if its effective mass is larger (Supplementary Table S2). Both our calculations and earlier studies^11^,^14^,^15^ based on DFT suggested that the effective mass of holes is larger than that of electrons. However, a recent study by using the quasiparticle self-consistent GW approximation (QSGW) shows that DFT poorly describes valence band dispersions of these perovskites^31^. The effective mass of holes obtained from SOC-DFT is twice that from QSGW. Since the hole effective mass from QSGW is even smaller, our conclusion that the hole mobility is larger than the electron mobility still holds, and the order of magnitude of the mobility does not change.

The thermopower is another important transport property of solids, which gives the charge carrier type and concentration of materials. By convention, its sign is negative for electrons and positive for holes. The logarithm decay of thermopower with the increasing carrier concentration has been observed in Fig. 3, irrespective of the charge carrier polarity. The maximum thermopower was −2.5 mV/K for electrons and 2.6 mV/K for holes, with little difference between cubic and tetragonal phases. The experimental value is −5 mV/K for single crystals of CH_3_NH_3_PbI_3_^20^.

In addition to the acoustic phonon scattering, there exist optical phonon scatterings whose contribution cannot be ruled out, since many low-frequency optical phonon modes of the PbI_3_ inorganic network have been identified^32^,^33^. Recently, we calculated the electron-phonon couplings in graphynes based on the density functional perturbation theory and the Wannier interpolation method^34^. There are low-frequency optical phonon modes in graphynes, but their electron-phonon couplings are small. In these 2D carbon materials, the longitudinal-acoustic phonon scattering is the dominant scattering mechanism over a wide range of temperatures. Such calculations can be applied to CH_3_NH_3_PbI_3_, but they are computationally demanding. Although we don’t know how much the optical phonon scattering contributes exactly, we expect that the acoustic phonon scattering is the most important scattering mechanism because the acoustic phonons have the lowest energy.

**Figure 4:**
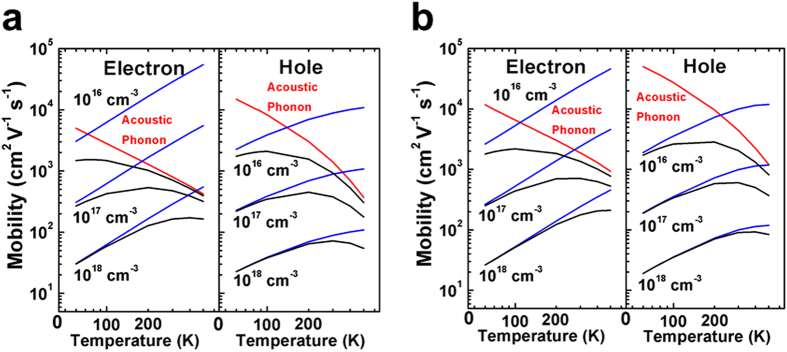
Temperature dependence of the charge carrier mobility limited by acoustic phonon and charged impurity scatterings. (**a**) Cubic CH_3_NH_3_PbI_3_ in the [100] direction. (**b**) Tetragonal CH_3_NH_3_PbI_3_ in the [001] direction. The free carrier density was taken as 10^14^ cm^−3^ for both electrons and holes, and the charged impurity density was set to 10^16^, 10^17^, and 10^18^ cm^−3^ respectively. The total mobility is shown in black, and that limited by acoustic phonon and charged impurity scatterings is shown in red and blue respectively.

During the low-cost solution process of materials, abundant intrinsic or extrinsic defects may form. Theoretical calculations have revealed that the intrinsic point defects do not generate gap states^17^,^18^,^19^, which explains the long diffusion length of charge carriers and the high open-circuit voltage of perovskites-based solar cells. However, defects can destroy the periodicity of a perfect crystal and serve as charge carrier scattering centers. In addition to the phonon scattering, charge carriers in perovskites are also subject to scatterings via Coulomb interactions with charged impurities or defects, such as the interstitial defects of organic anions or vacancy defects of lead, which have been shown to have low formation energies according to previous theoretical calculations^17^,^18^,^19^. The screened Coulomb potential leads to the scattering matrix element of the form^35^


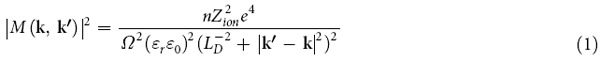


where *n* is the number of charged impurity or defect per unit cell, Ω is the volume of unit cell, *Z*_ion_ is the charge of impurity or defect ion, 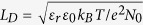 is the Debye screening length with *N*_0_ the free charge concentration, *ε*_r_ the relative permittivity of material and *ε*_0_ the dielectric constant of vacuum. The formula shows that the scattering matrix element is independent of the sign of charged impurities or defects, namely, both positively and negatively charged defects can scatter electrons or holes equally. Here, we set the absolute value of *Z*_ion_ to be uniformly one. The impurities are assumed to scatter charge carriers independently so that the scattering rate increases linearly with the impurity density. The impurity density was set to 10^16^, 10^17^ and 10^18^ cm^−3^ respectively, to show the impact of the impurity scattering on charge carrier transport. As can be seen from Fig. 3, the intrinsic mobility is independent of the carrier concentration, and the electrical conductivity increases linearly with the carrier concentration until 10^18^ cm^−3^. The charged impurity scattering instead, is dependent on the carrier concentration through the Debye screening length *L*_*D*_. However, we find that the mobility only change marginally with the carrier concentration between 10^10^ and 10^17^ cm^−3^ (Supplementary Fig. S4). From equation (1) we can infer that the screening starts to take effect when the Debye screening length becomes comparable to the lattice constant at higher carrier concentrations. The intrinsic carrier concentration of 10^10^ cm^−3^ has been obtained for solution-grown CH_3_NH_3_PbI_3_ single crystals from Hall effect measurements^4^, and the photogenerated carrier density of 10^17^ cm^−3^ was determined for CH_3_NH_3_PbI_3_ thin films under the laser pulse excitation intensity of 1 μJ cm^−2,^ 36. The carrier density related to the solar cell operation under AM 1.5 sun illumination was estimated to fall in the range of 2 × 10^13^ to 2 × 10^15^ cm^−3^ 37. Figure 4 showed that at the impurity concentration of 10^16^ cm^−3^ and the carrier density of 10^14^ cm^−3^, the acoustic phonon scattering dominates under the room temperature, while the charged impurity scattering starts to dominate at temperatures below 100 K. At the impurity concentration of 10^18^ cm^−3^, the charged impurity scattering dominates over the entire temperature range of 50–500 K. The mobility limited by the acoustic phonon scattering decreases with the temperature, while that limited by the charged impurity scattering increases with the temperature. Both display the power law temperature dependence. At the impurity concentration of 10^18^ cm^−3^ and 300 K, the hole mobility was 72.2 cm^2^ V^−1^ s^−1^ and 90.2 cm^2^ V^−1^ s^−1^ for the cubic phase in the [100] direction and the tetragonal phase in the [001] direction, and the electron mobility was 164 cm^2^ V^−1^ s^−1^ and 178 cm^2^ V^−1^ s^−1^ respectively. These values are in reasonable agreement with the experimental mobilities in CH_3_NH_3_PbI_3_ single crystals^3^. When the charged impurity scattering starts to dominate, the polarity of conduction is reversed with the electron mobility larger than the hole mobility. This is because the relaxation times of electrons and holes are close to each other at a certain defect density, but the effective mass of electrons is smaller.

## Discussion

To summarize, we have predicted the intrinsic and extrinsic charge transport properties for both cubic and tetragonal CH_3_NH_3_PbI_3_ based on first-principles calculations. We have found that the electron-acoustic phonon couplings in lead iodide perovskites are weak, and the deformation potential is comparable to that of single layer graphene. The intrinsic mobility limited by the acoustic phonon scattering is as high as a few thousands of cm^2^ V^−1^ s^−1^. However, intentional doping techniques have been widely used in both inorganic and organic semiconductor based devices including solar cells^38^. Theoretical calculations predicted that perovskites can be self-doped by defects engineering^17^,^19^. It was later experimentally demonstrated that CH_3_NH_3_PbI_3_ was either *n*- or *p*-doped by tuning the ratio of the two precursors for perovskite formation, PbI_2_ and CH_3_NH_3_I^34^. The perovskites films fabricated from the precursor ratio of 1.0 were shown to be heavily *n*-doped with an electron concentration of 2.8 × 10^17^ cm^−3^, and reducing the precursor ratio to 0.3 converted the films from *n*-type to *p*-type with a hole concentration of 4.0 × 10^16^ cm^−3^. The reduced carrier mobility with the increasing precursor ratio has been observed, which was ascribed to the increased dopant concentration in the perovskite films^39^. In those cases, scatterings by defects or impurities play a significant role in charge carrier transport. Our theoretical calculations show that at the charged impurity density of 10^18^ cm^−3^ and the carrier concentration lower than 10^17^ cm^−3^, charge carriers are scattered predominantly by charged defects or impurities in perovskites, and the room temperature mobility decreases to a few tens of and a hundred cm^2^ V^−1^ s^−1^ for holes and electrons respectively. The weak electron-phonon couplings and high intrinsic mobility of organolead triiodide perovskites promise that by defects and interface engineering, energy conversion efficiencies of perovskites-based solar cells can be enhanced with even simplified device structures^40^, such as that without a hole extracting layer^41^,^42^.

## Methods

### Structural optimization and band structure calculations

Density functional theory (DFT) with the Perdew-Burke-Ernzerhof (PBE)^21^ exchange-correlation functional was employed to optimize the strurtures. The projector-augmented wave method and a plane wave basis set with 400 eV cutoff were adopted as implemented in the Vienna ab-initio simulation package (VASP)^22^,^23^. Spin-orbit coupling (SOC) effect was considered throughout the calculations. The energy convergence criterion was set to 10^−5^ eV. The convergence criteria for structural optimization was reached when forces on every atom in every direction were smaller than 0.01 eV/Å. A **k**-mesh of 4 × 4 × 4 was used in the optimization, and that of 7 × 7 × 7 and 9 × 9 × 9 was subsequently used to obtain the converged charge density and density of states for tetragonal and cubic CH_3_NH_3_PbI_3_ perovskites.

### Boltzmann Transport theory

In the Boltzmann transport theory^30^, the deviation from the equilibrium Fermi-Dirac distribution of charge carriers in an external field is balanced by various scattering events with phonons and impurities, which help to restore the equilibrium distribution of charge carriers. In the relaxation time approximation, we can solve the Boltzmann transport equation to the first order of the external field, and arrive at the following expressions for the electrical conductivity 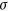 and Seebeck coefficient 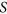:


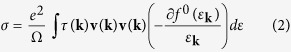



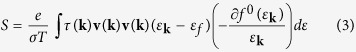


where 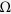 is the volume of unit cell, 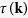 is the relaxation time, 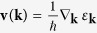 is the group velocity, 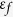 is the Fermi level, 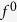 is the Fermi-Dirac distribution function. Similarly, the charge carrier mobility can be written as


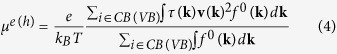


in which the group velocities can be calculated on a dense **k**-mesh from first-principles, which is 31 × 31 × 31 for the tetragonal and 41 × 41 × 41 for the cubic CH_3_NH_3_PbI_3_.

According to the Mathiessen’s rule, by assuming that various scattering mechanisms are independent of each other, the total relaxation time can be expressed as


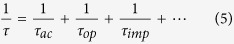


in which 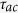 is the relaxation time due to the acoustic phonon scattering, 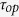 and 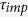 represent the relaxation times due to the optical phonon scattering and ionic impurity scattering, respectively. In case of the acoustic phonon scattering, the phonon energy is so small that the scattering is considered elastic. By applying the Fermi’s golden rule, the relaxation time is further written as


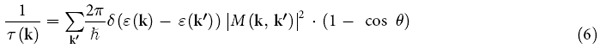


where 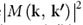 is the scattering matrix element, which has different forms for different scattering mechanisms. 
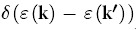
 is the Dirac delta function, 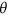 is the scattering angle between wave vectors of two electronic states denoted by 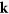 and 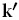. The acoustic phonon scattering in the long wavelength limit is modeled by the DP theory, with the scattering matrix element taking the form


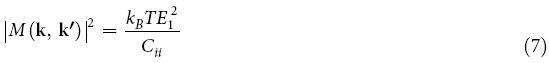


where 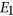 is the deformation potential constant and 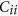 is the elastic constant. 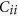 was obtained by the parabolic fitting of the total energy 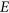 of a unit cell with respect to the dialation 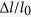 via 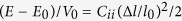, and 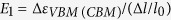 was obtained by the linear fitting between the energy shift of VBM and CBM and the dilation for holes and electrons respectively. Here we assume that the deep energy level barely changes during dilation, which was first proposed by Wei and Zunger^28^, and take the deep energy level localized on the organic cation as a reference to calibrate the shift of VBM and CBM during dilation.

We use the Brooks-Herring approach^35^ to model the scattering of charge carriers by ionic impurities or defects. The screened Coulomb potential of an ionic impurity takes the form


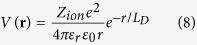


where 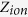 is the charge of the ionic impurity, 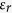 is the relative dielectric constant and 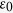 is the vacuum dielectric constant. 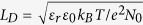 is called the Debye screening length and 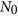 is the free charge carrier density. The scattering matrix element has been given in equation (1) as 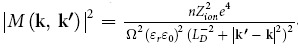. The relative dielectric constant was set to 6.5 according to ref. 43.

## Additional Information

**How to cite this article**: Zhao, T. *et al.* Intrinsic and Extrinsic Charge Transport in CH_3_NH_3_PbI_3_ Perovskites Predicted from First-Principles. *Sci. Rep.* **6**, 19968; doi: 10.1038/srep19968 (2016).

## Supplementary Material

Supplementary Information

## Figures and Tables

**Table 1:** Elastic constant, deformation potential, average relaxation time, mean free path, and mobility of holes and electrons for cubic and tetragonal CH_3_NH_3_PbI_3_.

Axis		Cubic			Tetragonal		
		*a*	*b*	*c*	*a*	*b*	*c*
*C*_*ii*_ (Gpa)		7.5	21.5	22.1	19.3	19.2	10.3
*E*_1_ (eV)	e	4.9	2.0	2.0	1.1	0.4	4.3
	h	2.2	0.6	0.6	1.5	1.2	1.3
μ(cm^2^ V^−1^ s^−1^)	e	800	572	572	2554	2494	1876
	h	1432	2156	2157	7176	7310	4412
*τ*(ps)	e	0.12			0.33		
	h	0.67			1.87		
*l* (nm)	e	21.6			67.3		
	h	90.8			284		
