# Erratum: Intrinsic and Extrinsic Charge Transport in CH_3_NH_3_PbI_3_ Perovskites Predicted from First-Principles

**DOI:** 10.1038/srep46200

**Published:** 2017-04-05

**Authors:** Tianqi Zhao, Wen Shi, Jinyang Xi, Dong Wang, Zhigang Shuai

Scientific Reports 6: Article number: 1996810.1038/srep19968; published online: 01 29 2016; updated: 04 05 2017

The original HTML version of this Article listed an incorrect volume number. This has now been corrected in the HTML version; the PDF version was correct at the time of publication.

