# Supplementary material for: Intrinsic and Extrinsic Charge Transport in CH3NH3PbI3 Perovskites Predicted from First-Principles
Source: Sci Rep. 2016 Jan 29;7:19968. doi: 10.1038/srep19968 (PMC4731798; doi:10.1038/srep19968)
Supplement: Supplementary Information [file srep19968-s1.pdf]

Supplementary Information

Intrinsic and Extrinsic Charge Transport in $CH_3NH_3PbI_3$ Perovskites Predicted from First-Principles

Tianqi Zhao,[1] Wen Shi,[1] Jinyang Xi,[1] Dong Wang,[*,1] Zhigang Shuai[*,1,2,3]

[1]MOE Key Laboratory of Organic OptoElectronics and Molecular Engineering, Department of Chemistry, Tsinghua University, Beijing 100084, P. R. China

[2]Key Laboratory of Organic Solids, Beijing National Laboratory for Molecular Science (BNLMS), Institute of Chemistry, Chinese Academy of Sciences, Beijing 100190, P. R. China

[3]Collaborative Innovation Center of Chemistry for Energy Materials, Xiamen University, 351005 Xiamen, China

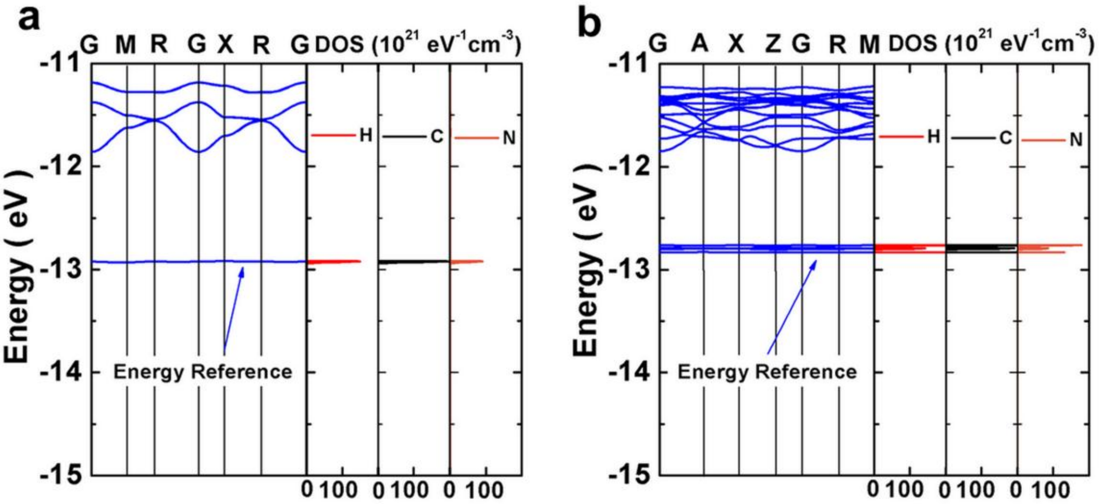

Supplementary Figure S1. Energy reference for the VBM and CBM calibration during straining of cubic (a) and tetragonal (b) $CH_3NH_3PbI_3$.

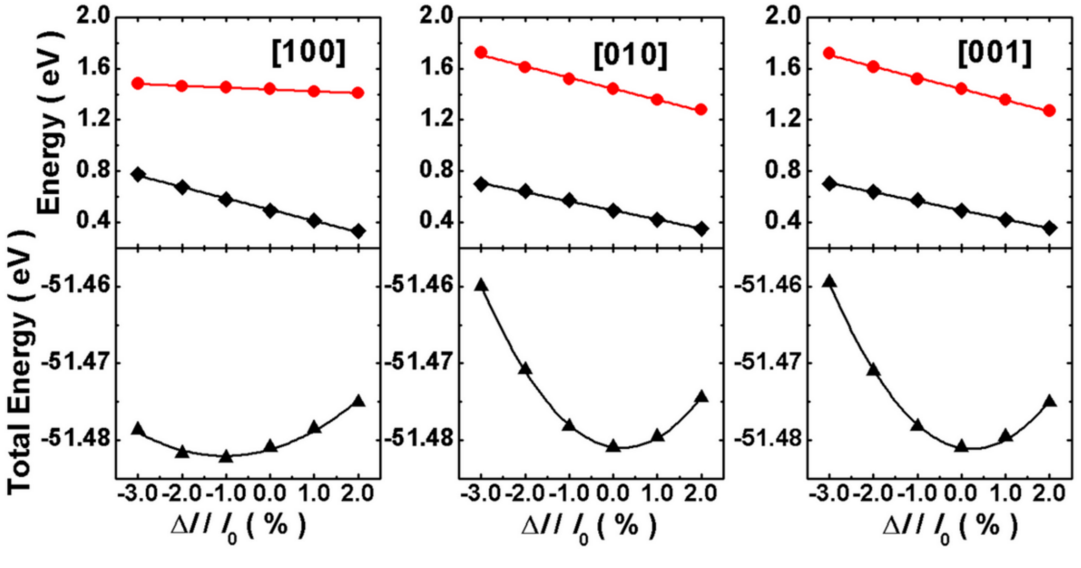

Supplementary Figure S2. Energy shift of the CBM and VBM with respect to dilation (upper panel) and total energy change with dilation (lower panel) along [100], [010] and [001] directions for cubic $CH_3NH_3PbI_3$.

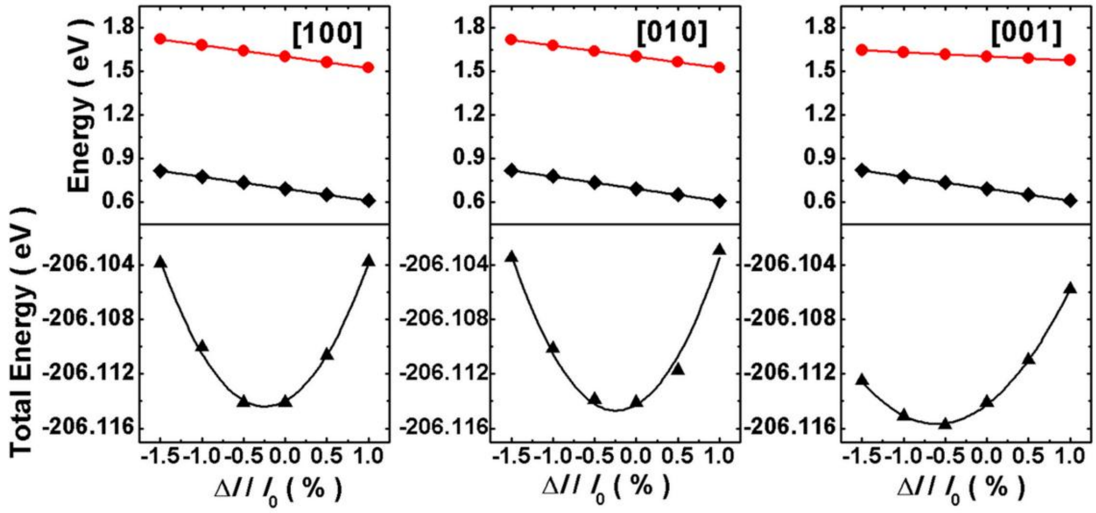

Supplementary Figure S3. Energy shift of the CBM and VBM with respect to dilation (upper panel) and total energy change with dilation (lower panel) along [100], [010] and [001] directions for tetragonal $CH_3NH_3PbI_3$.

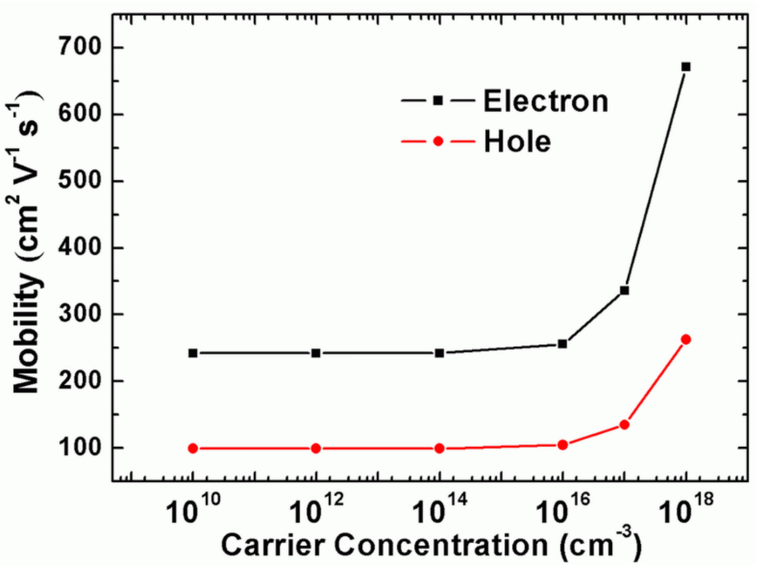

Supplementary Figure S4. Mobility of tetragonal $CH_3NH_3PbI_3$ perovskites limited by charged impurity scattering as a function of free carrier concentration at an impurity density of 10^{18} cm^{-3}.

Supplementary Table S1. Structural parameters of cubic and tetragonal $CH_3NH_3PbI_3$.

		a (Å)	*b* (Å)	*c* (Å)	α	β	γ
Cubic	Calc.	6.67	6.44	6.44	89.76	90.02	90.02
	Expt.[a]	6.31	6.31	6.32	90.0	90.0	90.0
Tetragonal	Calc.	9.11	9.07	12.91	90.25	89.25	88.32
	Expt.[a]	8.85	8.85	12.64	90.0	90.0	90.0

[a]Ref. S1.

Supplementary Table S2. Effective mass m^*/m_e of electrons and holes in cubic and tetragonal $CH_3NH_3PbI_3$. 1 and 2 represent bands with opposite spins.

		VB(2)	VB(1)	CB(1)	CB(2)
Cubic	R-M	0.60	0.28	0.16	0.30
	R-G	0.52	0.26	0.17	0.28
	R-X	0.55	0.31	0.18	0.32
Tetragonal	G-M	0.52	0.28	0.15	0.23
	G-Z	0.45	0.43	0.26	0.27
	G-X	0.31	0.24	0.16	0.20
	G-A	0.50	0.28	0.15	0.24
	G-R	0.36	0.26	0.17	0.23

Supplementary Table S3. Relaxation times of electrons and holes in cubic and tetragonal $CH_3NH_3PbI_3$ subject to impurity and acoustic phonon scatterings at 300 K. The impurity density is taken as 10^{16}, 10^{17} and 10^{18} cm^{-3} respectively. The free carrier concentration is 10^{14} cm^{-3}.

τ (ps)		Cubic		Tetragonal	
		Electron	Hole	Electron	Hole
Ionized Impurity	10^{18} cm^{-3}	0.017	0.019	0.016	0.018
	10^{17} cm^{-3}	0.17	0.19	0.16	0.18
	10^{16} cm^{-3}	1.67	1.95	1.59	1.83
Total	10^{18} cm^{-3}	0.011	0.017	0.013	0.017
	10^{17} cm^{-3}	0.042	0.10	0.065	0.13
	10^{16} cm^{-3}	0.082	0.30	0.16	0.51

Supplementary Table S4. Mobilities of electrons and holes in cubic $CH_3NH_3PbI_3$ subject to impurity and acoustic phonon scatterings at 300 K. The impurity density is taken as 10^{16}, 10^{17} and 10^{18} cm^{-3} respectively. The free carrier concentration is 10^{14} cm^{-3}.

μ (cm^2 V^{-1} s^{-1})		electron			hole		
		a	*b*	*c*	*a*	*b*	*c*
Ionized Impurity	10^{18} cm^{-3}	288	172	172	88.7	151	151
	10^{17} cm^{-3}	2878	1716	1718	887	1506	1506
	10^{16} cm^{-3}	28781	17157	17175	8871	15062	15065
Total	10^{18} cm^{-3}	164	101	101	72.2	119	119
	10^{17} cm^{-3}	473	303	303	377	592	591
	10^{16} cm^{-3}	708	476	476	927	1399	1399

Supplementary Table S5. Mobilities of electrons and holes in tetragonal $CH_3NH_3PbI_3$ subject to impurity and acoustic phonon scatterings at 300 K. The impurity density is taken as 10^{16}, 10^{17} and 10^{18} cm^{-3} respectively. The free carrier concentration is 10^{14} cm^{-3}.

μ (cm^2 V^{-1} s^{-1})		electron			hole		
		a	*b*	*c*	*a*	*b*	*c*
Ionized Impurity	10^{18} cm^{-3}	184	185	242	158	161	99.2
	10^{17} cm^{-3}	1844	1853	2423	1577	1613	992
	10^{16} cm^{-3}	18442	18534	24228	15768	16132	9918
Total	10^{18} cm^{-3}	139	139	178	142	145	90.2
	10^{17} cm^{-3}	590	585	711	930	949	605
	10^{16} cm^{-3}	1294	1261	1365	3086	3150	2050

Supplementary References

S1. Stoumpos, C. C., Malliakas, C. D. & Kanatzidis, M. G. Semiconducting tin and lead iodide perovskites with organic cations: Phase transitions, high mobilities, and near-infrared photoluminescent properties. *Inorg. Chem.* **52,** 9019-9038 (2013).
